# The emerging modern face of mood disorders: a didactic editorial with a detailed presentation of data and definitions

**DOI:** 10.1186/1744-859X-9-14

**Published:** 2010-04-12

**Authors:** Konstantinos N Fountoulakis

**Affiliations:** 1 Third Department of Psychiatry, School of Medicine, Aristotle University of Thessaloniki, Greece

## Abstract

The present work represents a detailed description of our current understanding and knowledge of the epidemiology, etiopathogenesis and clinical manifestations of mood disorders, their comorbidity and overlap, and the effect of variables such as gender and age. This review article is largely based on the 'Mood disorders' chapter of the Wikibooks *Textbook of Psychiatry *http://en.wikibooks.org/wiki/Textbook_of_Psychiatry/Mood_Disorders.

## Background

The ancient Greeks Hippocrates (460 to 357 BC), Galen (131 to 201 AD) and Areteus from Kappadokia introduced the terms melancholia and mania. Hippocrates was the first to describe melancholia, which is the Greek word for 'black bile', and simultaneously postulated a biochemical origin according to the scientific frame of that era, linking it to Saturn and the autumn. The term 'mania' was used to describe a broad spectrum of excited psychotic states. Soranus from Ephesus was the first to describe mixed states. Manic depressive illness has also been known since antiquity and Aretaeus of Cappadocia (2nd century AD) is considered to be the first to strongly connect melancholy with mania and make a description of manic episodes very close to the modern approach, including psychotic features and seasonality. Another interesting element in the theories that emerged during antiquity was the concept of temperament, which was originally based on harmony and balance of the four humours, of which the sanguine humour was considered to be the healthiest but also predisposing to mania. The melancholic temperament was linked to black bile and was considered to predispose to melancholia. Since the time of Aristotle (384 to 322 BC), the melancholic temperament was linked to creativity.

Later, the Arab scholars dominated (Ishaq Ibn Imran, Avicenna and others) in particular during the 10th and 11th centuries AD. In 1621, Robert Burton wrote the first English language text, the *Anatomy of Melancholy*. Later, the works of Jean-Philippe Esquirol (1772 to 1840), Benjamin Rush (1745 to 1813), Henry Maudsley (1835 to 1918), Jean-Pierre Falret (1794 to 1870) and Jules Gabriel Francois Baillarger (1809 to 1890) finally established the connection between depression and mania. Eventually, Emil Kraepelin (1856 to 1926) established manic depressive illness as a nosological entity by separating it from schizophrenia on the basis of heredity, longitudinal follow-up and a supposed favourable outcome. In contrast, today the suboptimal outcome of mood disorders is well documented, especially in relationship to younger age of onset and to alcohol and substance abuse. Suicide is another major concern, since up to 75% of patients who commit suicide have some type of mood disorder. Thus, recent research data have tended to radically reshape our definition and understanding of mood disorders.

Combined, affective disorders are the most disabling neuropsychiatric conditions and one of the four leading disability causes according to the World Health Organization (WHO), which ranked psychiatric disorders as the most disability-inducing cause worldwide; more disabling than cancer and cardiovascular diseases and equal to injuries from all causes (Appendix 1) [1]. The present article attempts to summarise our current concept and understanding of mood disorders. A more extensive approach can be found in the 'Mood disorders' chapter of the Wikibooks *Textbook of Psychiatry *(free full text access at http://en.wikibooks.org/wiki/Textbook_of_Psychiatry/Mood_Disorders), on which the current article is based to a significant degree.

## Epidemiology

Unipolar major depressive disorder (U-MDD) as defined by the Diagnostic and Statistical Manual of Mental Disorders, fourth edition text revision (DSM-IV-TR) is reported to be the most common mood disorder [2], with an overall prevalence of 4.7% for males and 6% for females. Its annual incidence is around 1.59%. Beyond the DSM definition, depression of any type might affect up to 10% to 25% of females and 5% to 12% of males at some time during their lives, with the rates varying widely and depending on ethnic background, residential area, gender, age, social support and general somatic health status [3-5].

Sometimes people experience a single mood episode in life, but around half of those experiencing an episode will experience more in the future and the likelihood after the second episode is to experience a third within a decade or so. One-third of patients will recover within the first 2 to 3 months, another third will need 6 to 8 months and around 15% of patients will not have recovered after 2 years; they are likely to experience a chronic course of disorder [6-13]. Moreover, in spite of treatment, disability rates are high and suicide occurs in about 15% of patients, especially in men [14-16]. With regard to bipolar disorder (BD), It has been suggested that it has a prevalence of around 1% (0.4% to 1.6%), however today we know that the true prevalence depends on the definition and extent of subthreshold bipolar cases, pseudounipolar patients and personality disorders (PDs), especially 'borderline personality disorder', that are included under the umbrella of the 'bipolar spectrum' or under 'unipolar depression'. DSM-IV-TR BD types É and ÉÉ have a combined prevalence rate of up to 3.7%. The literature on the lifetime prevalence of BD suggests an overall rate of 3% to 6.5%, including a wider spectrum of bipolarity in comparison to the DSM-IV-TR definition [17-19].

The data concerning the risk factors for MDD are inconclusive. The few available community-based studies suggest that younger age, low social class, and negative and stressful life events linked to the family were associated with increased risk of new onset of depression [20]. Other studies suggest that female gender [21-24], marital status, family history of depression, suicide and alcoholism, early childhood abuse [25], specific personality features (introversion, worry, dependency and interpersonal sensitivity) [26-30], life events (especially loss and bereavement), chronic stress (financial, family and interpersonal difficulties), and daily hassles [31-33] constitute important risk factors, with age playing a complex role [34]. MDD has an average age of onset between 20 and 40 years while BP appears most frequently in the early 20 s [22]. It seems that the genetic loading is stronger for BD in comparison to U-MDD [35].

Studies have shown that in nearly all countries worldwide, women have nearly double the rate of depression than men, although this is not well documented in non-industrialised cultures [36]. The National Comorbidity Survey reported that 6% of females vs 3.8% of males had a current depressive episode and that 21.3% of women vs 12.7% of men had a lifetime experience of a depressive episode [37]. The rates for BD are similar, however, suggesting this difference concerns only unipolar depression. Interestingly, the female to male ratio increases as we move from bipolar I to bipolar II and to unipolar depression, suggesting that this ratio increases as the depressive component becomes dominating. A second finding suggests that women with less social support and those women experiencing social stressors might be at the greatest risk of developing depression. However, there is no significant gender difference concerning the risk of recurrence, thus suggesting that gender is among the risk factors for initiating depressive symptoms but not among those determining the course and outcome. This higher risk for females is present from around age 20 upwards until the early 30 s, and the rates of first onset before (childhood and adolescence) or after that age (middle age, older) are similar for both sexes [38,39].

It is possible that the profile of depression among men differs from that among women. Males are typically characterised by a constellation of atypical symptoms including irritability, aggressiveness, acting out, antisocial behaviour and alcohol abuse, alexithymia and reduced impulse control and stress tolerance, and this pattern seems to be related to central serotonin deficiency and hypercortisolaemia. Men seem to be incapable of asking for help, and the atypicality of their clinical picture often leads to rejection or misdiagnosis in the healthcare system, resulting in underdiagnosis and undertreatment that may explain the paradoxical fact that men are only half as often depressed but are five times more likely to commit suicide than females (for example, as seen in Sweden) [40-42].

It seems highly unlikely that there is a single, sex-related factor responsible for the difference. Endocrine changes and differences were the target of research without convincing results. The role the female reproductive system might play in mental health is still controversial. The fact that the gender difference is not obvious until puberty, and disappears after menopause, supports the idea that there is something specific connecting female biology to mood disorders. A more advanced approach suggests that this biology is not a risk factor *per se*; on the contrary, it could be responsible for an increased vulnerability to stressors, thus indirectly leading to depression, especially considering the fact that women are more likely to experience stressful and even threatening life events and are at a higher risk of early sexual abuse and current spousal abuse [43,44]. They also might use oral contraceptives, and often experience mood disorders temporally related to their sexual identity (for example, mood disorders of premenstrual or postpartum onset). Additionally, almost all societies have designated different, unequal roles for women.

However, since no conclusive data are available so far, it is necessary to consider the possibility that men and women share similar rates of depression but they express depression in different ways, and the resulting different rates are in reality a methodological artefact. In this case, it's reasonable to suggest that different cognitive coping styles between men and women could be responsible for these results and it may be women are more likely to be diagnosed with depression because they seek professional help more often for their depressive symptoms, and they are more sensitive to negative relationships [45]. It is believed that men might react to emotional distress by trying not to think about it, while women are more likely to ruminate over their problems [46-48]. In this sense, women are more likely to report depressive symptoms due to marital problems than men. This could at least partially be socioculturally determined, or imposed, since it is reported that the depressed female students who reached out to their friends were met with concerned and nurturing reactions, while in contrast, depressed male students who did the same faced social isolation and often direct rejection, even hostility [49,50]. While married, divorced, and separated women were more likely to be depressed than men, widowed men were more likely to be depressed than women and unmarried men and women shared similar rates of depression [51].

Another possibility is that in men, but not in women, alcohol abuse could mask an underlying depressive disorder and could account for the difference in rates. This opinion is derived from the observation that alcohol abuse and mood disorders are often inherited in the same family [52].

## Etiopathogenesis

Today, most mood disorders experts agree that mood disorders have both endogenous and exogenous components and, in most patients, they are both present. After the historical dualism suggested by Rene Descartes in the 17th century, it is only as recently as the early 20th century that Adolf Meyer used the term 'psychobiology' to emphasise that psychological and biological factors interact in the development of mental disorders. The biopsychosocial model has been proposed by Engel [53,54], and provides a non-specific but inclusive theoretical framework in order to host all variables suggested by various approaches to cause depression.

## Social stressors

Although lay people and much of psychological theory attribute mood disorders to adverse life events, there are several studies which dispute the role stressful life events play in the development or the course of depression [55,56]. However, the sensitisation of stress-responsive neurobiological systems as a possible consequence of early adverse experience has been more solidly implicated in the pathophysiology of mood and anxiety disorders. A history of childhood abuse *per se *may be related to increased neuroendocrine stress reactivity, which is further enhanced when additional trauma is experienced in adulthood [25]. In this sense, depressed patients were reported to have higher perception of day to day stressors (hassles), reduced perception of uplifting events, excessive reliance on emotion-focused coping strategies, and diminished quality of life in comparison to controls. Among depressed patients the hassles, coping styles and some elements of quality of life were related to symptom severity, as well as treatment resistance [57]. The question that arises is whether this is a true fact or whether these patients (which have higher personality psychopathology and interpersonal rejection sensitivity) tend to over-report life events [41].

Thus, many authors insist that psychosocial factors are relatively unimportant in the subsequent course of severe and recurrent depressions, in contrast to their contribution to the onset of such depressions and subsequent outcome of milder depressions [58,59].

## Psychological models of mood disorders

There are a number of psychological models that have been proposed over the last 100 years to explain the pathogenesis of depression. The most important are the following:

### Aggression turned inward

This was proposed by Sigmund Freud and Karl Abraham on the basis of a 'metaphor' from physics to psychology ('hydraulic mind'). According to this model, during the oral phase (that is, during the 12th to 18th months of life) disturbances in the relationship between the infant and the mother establish a vulnerability to develop depression. Then, during the adult life, a real or imaginary loss leads to depression as the result of aggressive impulses turned inward and directed against the ambivalently loved internalised object that had been lost. The aim of that turned-inward aggression was supposed to be the punishment of the love object, which fails to fulfil the patient's need to be loved. It is therefore accompanied by guilt, which can lead to suicidal behaviour. Later, other authors proposed somewhat different versions of this model. The drawbacks of this model include that it represents a relatively closed circuit independent of the outside world, while the clinical fact is that many depressed patients openly express anger and hostility to others that is reduced after treatment, and that there is no evidence supporting the concept that expressing anger outwardly has a therapeutic effect in the treatment of clinical depression.

### Object loss

This term refers to traumatic separation from significant objects of attachment. However according to empirical research data, only a minority of no more than 10% of people experiencing bereavement will eventually manifest clinical depression. Thus, the model includes two steps; an early one that includes significant loss during childhood, creating a vulnerability that, during the second step of significant loss during adult life, leads to clinical depression. This model better fits the data in comparison to the aggression turned inward model and has some support from studies on primates, although the latter point to a broad psychopathology rather than specifically depression.

### Loss of self-esteem

Depression is considered to originate from the inability of the ego to give up unattainable goals and ideals, resulting in a collapse of self-esteem. This model suggests that the narcissistic injury that destroys the patient's self-esteem comes from the internalised values of the ego rather than the hydraulic pressure deriving from the id as proposed by the aggression turned inward model. In this sense the loss of self-esteem has a sociocultural and existential dimension and thus this theory is testable to a significant extent. The drawback of this theory is that both persons with low and high self-esteem can develop depression or mania without any significant differences among them.

### Cognitive model

The cognitive model was developed by Aaron Beck, and suggests that thinking in a negative way is the core of clinical depression. According to this, depression is conceptualised in the sense of the 'cognitive triad'. This triad proposes that patients conceive the self, the environment and the future in a negative depressive way (helplessness, negative and hopelessness). In the core there seems to be bias of the person in the way of thinking and interpreting, which results in a profound negative attributional style (mental schemata) that is considered to be global, internal, and stable. The bias in the way of thinking is because of overgeneralisation, magnification of negative events with a simultaneous minimisation of positive events, arbitrary inference, and selective abstraction. Systematic errors in thinking allow the persistence of negative schemas despite contradictory evidence. The major drawback of this model is the fact that it is based on retrospective observations of depressed patients, thus the negative triad could be simply subclinical manifestations of depression and not the cause of it. The major advantage is that it led to the first testable and practical psychotherapeutic approach, which seems to be effective in a specific subgroup of patients.

### Learned helplessness model

This model is based on animal experiments and proposes that the depressive attitude is learned during past situations in which the person was not able to terminate or avoid undesirable or traumatic events. However it seems that the learned helplessness paradigm is more general and refers to a broader mental condition (for example, social behaviour, post-traumatic stress disorder, and so on). It seems that past events could shape a personality profile that includes passivity, lack of hostility, and self-blame. However this line of thinking could lead to the notion that depression and the behaviours accompanying it should be considered to be a result of a masochistic lifestyle with manipulative behavioural patterns in order to handle interpersonal issues. Further, recent animal research has implicated the importance of genetic factors in the vulnerability to learning to behave helplessly.

### Depression and reinforcement

According to the reinforcement model, behaviours characteristic for depression develop because of a lack of appropriate rewards and receipt of non-contingent rewards. This theory bridges personality, low self-esteem and learned helplessness with the human social environment; however, it seems more appropriate for the interpretation of social issues than clinical depression. A psychotherapeutic approach aiming to improve the patient's social skills is based on this theory.

## Psychological theories of mania

Most theories view manic symptoms as a defence against an underlying depression, with the use of a number of defence mechanisms such as omnipotence, denial, idealisation, and contempt. In this sense, the euphoric state of the patient is understood as a tendency to extinguish any unpleasant aspects of reality and to disregard the problems of reality, even if the situation is tragic. Thus, mixed episodes are easily psychodynamically understood, since manic elements seen in depressed patients are considered to be defences.

## Biological models of mood disorders

Data from animal experiments and models have implicated the limbic-diencephalic brain in mood disorders and more specifically neurons containing serotonin and noradrenaline. Historically the monoamine deficiency hypothesis is based on data from the study of cerebrospinal fluid (CSF) metabolites. According to this theory, there is a monoamine deficiency, especially norepinephrine (NE), in depression. Later, studies illustrated that this theory should also include serotonin (5-hydroxytryptamine; 5-HT), leading to a broader theory regarding neurotransmission disorder in the central nervous system (CNS) [60-62]. Further, the cholinergic-noradrenergic imbalance hypothesis [63] included acetylcholine in a broader model for mood disorders. More complex models include state changes (depending on the polarity of the mood episode) in the excitatory amino-acid function in specific areas of the cortex [64].

However, in spite of decades of extensive research there is no definite proof for either a deficiency or an excess of either the quantity or the overall functioning of biogenic amines in specific brain structures. Even when these abnormalities were documented, it has been shown that they are neither necessary nor sufficient for the occurrence of mood disorders. In contrast, it seems that the neurotransmitter disorders recognised up to the present day refer to a broader behavioural dysfunction, which includes behavioural disinhibition, obsessive-compulsive symptoms, anxiety, eating disorders and substance and alcohol abuse, as well as personality disorders. This is not peculiar since most classic animal models are in essence post-traumatic stress models and most biological psychoendocrinological markers are markers of stress-related somatic reactions. Recent research has explored disturbances at the level of second messengers and DNA function with variable success, but no definite conclusions.

A number of biological markers have been developed so far but no single one has proved good enough for use in clinical practice. The dexamethasone suppression test (DST) has been widely used for the study of hypothalamus-pituitary-adrenal (HPA) axis disorders in patients with depression [65-67]. It requires the oral administration of 1 mg dexamethasone (a synthetic glucocorticoid) at 23:00 on day 1 and the assessment of cortisol levels at the same time, and at 08:00, 16:00 and 23:00 on day 2. A cortisol value of 5 μg/dl for at least one measurement on day 2 is considered to be the cut-off point between normal (suppressors) and pathological (non-suppressors). Longer protocols requiring higher dosages for dexamethasone and a 24-hour assessment have also been suggested. The test presents a 67% sensitivity and 96% specificity in the diagnosis of melancholy in psychiatric inpatients. Abnormal DST results also relate to the presence of psychotic symptoms in both unipolar and bipolar patients [68,69]. The results of the up to date research efforts report that DST presents results that are probably related with the severity of depression and the patient's family history.

Other psychoendocrinological markers are the thyroid-stimulating hormone (TSH) stimulation test (blunted TSH response to thyrotropin-releasing hormone) [70,71], the fenluramine and D-fenfluramine challenge tests [72-81] that are supposed to reflect central serotonin activity (administration of 30 mg of the D-fenfluramine orally and measurement of prolactin plasma levels at the baseline after 60, 120, 180, 240 and 300 minutes after the administration), blunted growth hormone (GH) response to the α2-adrenergic receptor agonist clonidine (an index of noradrenergic dysregulation) and others. One non-endocrinological marker is based on electroencephalogram (EEG) readings and concerns the observation that depressed patients are phase advanced in many biological rhythms, especially concerning the latency to the first rapid eye movement (REM) in sleep (shortened REM latency) [82].

A possible comprehensive model could suggest that mood patients have a deficit in the adequate mobilisation of neurotransmitters when facing continued or repeated stress, and as a result, through a 'kindling' effect [71,83-88], the mood change is intense, prolonged and not self-limited, and tends to be triggered by progressively unimportant events and finally automatically. Thus it is expected that an early application of treatment with antidepressants and psychotherapy could prevent neuroplastic changes and the long-term worsening of the clinical course.

The data from family and twin studies argue strongly for the familial nature of mood disorders [89,90]. However, so far the mode of genetic transmission remains elusive. Several studies have focused on a functional polymorphism in the promoter region of the serotonin transporter gene (HTTLPR), which is supposed to moderate the influence of stressful life events on depression and the brain-derived neurotrophic factor (BDNF), which is supposed to exert a prophylactic effect against neuronal toxicity induced by stress [91-93]. The most likely model is a multifactorial threshold model. The twin data suggest that genes account for 50% to 90% of the aetiology of mood disorders [94].

## Clinical manifestations

The onset of mood episodes could be acute, or insidious and arise from a low-grade, intermittent, and protracted mood substrate that could resemble a dysthymic or cyclothymic state or even personality features. These mood states could also prevail during the interepisode period, and might give rise to low quality of life, interpersonal conflicts and significant global disability [95]. However, both dysthymic and cyclothymic disorders are recognised by contemporary classification systems as separate diagnostic entities and often do not lead to the manifestation of a full-blown mood episode.

Bipolar disorders consist of at least one hypomanic, manic, or mixed episode. Mixed episodes represent a simultaneous mixture of depressive and manic or hypomanic manifestations. Although a minority of patients experience only manic episodes, most bipolar disorder patients experience episodes of both polarities.

The classical definition of BD suggests that this disorder is characterised by the presence and alteration of manic and depressive episodes, with a return to premorbid level of functioning between the episodes and a favourable outcome in comparison to schizophrenia [96]. Today we know that this is not always the case [97]. The Kraepelinian concept largely corresponds to BD type I (BD-I) according to DSM-IV-TR [98]. Typically, BD-I starts before the age of 40. Frequently the correct diagnosis is made after several years because the first episode is psychotic-like or depressive and the diagnosis is made only after a manic or mixed episode emerges. Another type, BD-II, is officially recognised as a bipolar illness subtype and it is characterised by the presence of hypomanic instead of manic episodes. However, it is important to note that according to DSM-IV-TR [98] hypomania is defined mainly in terms of a shorter duration of the episode. BD-II is more prevalent than BD-I. An additional problem for diagnosis is that patients usually experience hypomania as a recovery from depression, and almost always as a pleasant egosyntonic mood state.

Depressive episodes are considered to be the second diagnostic pillar of BD. However, in contrast to manic episodes that lead to the diagnosis of BD immediately, depressive episodes pose a dilemma to the clinician whether he faces unipolar depression or BD. This is an important dilemma to solve since the treatment is different for these disorders. However, it has been estimated that more than half of patients originally manifesting a depressive episode will turn out to be bipolar in the next 20 years [99]. Unipolar-depressed patients who later 'convert' to BD over time, as well as bipolar depressives, more frequently manifest 'atypical' depressive features (hypersomnia, hyperphagia, leaden paralysis, long-term interpersonal rejection sensitivity) [100], psychomotor retardation, psychotic features, pathological guilt and mood lability. BD patients also tend to have an earlier age of onset, more prior episodes of depression, shorter depressive episodes, and family history of BD [101,102]. Family history of BD is a strong predictor of bipolarity even in children and adolescents [103]. DSM-IV-TR recognises atypical features of depression [104-106]. This depressive subtype includes the presence of personality-like features such as long-term interpersonal rejection sensitivity, and somatic symptoms such as reverse vegetative signs, hypersomnia, increased appetite, weight gain and leaden paralysis. There is strong evidence linking atypical depression to BD-II [107]. The clinical features more common in bipolar depression are summarised in Appendix 1.

Mixed episodes are also considered to be part of the BD picture, and according to DSM-IV-TR are defined as the coexistence of both depressive and manic symptoms to the extend the criteria for both a manic and a depressed episode are fulfilled [108]. Alterations in mood characterise several other DSM disorders that have a bipolar character. These include cyclothymic disorder and borderline personality disorder. However, there is a constellation of types of affective episodes that are not part of the official classification and they are so prevalent in real life clinical practice that many authors consider them to be the rule rather than the exception.

Sometimes there is an admixture of a number of manic and depressive symptoms in a combination that does not fulfil the specific DSM criteria for a manic, depressive or mixed episode, thus the only possible diagnosis is that of a not otherwise specified (NOS) mood episode [109,110].

Often the manic symptoms can go unnoticed by the clinician because instead of being hyperthymic, the mood is irritable and it is diluted in the presence of depressed thought content and suicidal ideation, leading the clinician to the diagnosis of anxious or agitated depression, or worse, a personality disorder, instead of a mixed or mixed NOS mood episode. Frequently, this irritable mood can lead the person to manifest aggressive behaviour, especially if confronted or rejected while having grandiose and paranoid delusions, and these patients are maybe the most aggressive seen in the emergency room [111,112].

There is evidence that a development of an excited/irritable state could happen when antidepressants, especially dual action ones, are used. Many patients will not develop a classic manic episode in response; many will either develop a full-blown mixed episode or more likely a DSM-subthreshold mixed NOS episode with the presence of a small number of manic symptoms in combination with depression, especially agitation, and this state could persist and worsen if more aggressive antidepressant treatment is tried.

The term 'rapid cycling' refers to patients with at least four mood episodes in a year. It seems that females are more often rapid cyclers, as well as of higher social class. In essence, these patients tend to be symptomatic most of their life and are considered to be refractory to lithium. The diagnosis can be elusive for prolonged periods of time and the patients can receive the misdiagnosis of a personality disorder or cyclothumia. Treatment is based on a complex, delicate and difficult to design multiple pharmacotherapy, which includes atypical antipsychotics, anticonvulsants and even antidepressants, although the latter are believed to induce rapid cycling [113].

Psychotic features are common in bipolar patients and may include delusions or hallucinations of any type. They can either be congruent or non-congruent, and both could occur in the context of a mood disorder. In order to make the diagnosis of schizoaffective disorder according to DSM-IV-TR, there must have been a psychotic episode in the absence of prominent mood symptoms. However, in the International Classification of Diseases, 10th edition (ICD-10), this diagnostic boundary is vague and differential classification is often difficult.

Alcohol and substance abuse are very common problems in BD. Drug abuse may precipitate an earlier onset of BD-I in those who already have a familial predisposition for mania. Alcohol abuse could be present in more than half of patients. It seems that frequently this represents self-medication efforts and abuse is particularly problematic during adolescence and early adulthood. During this age period, substance and alcohol abuse might not only suppress symptoms but also enhance specific desired activities (for example, high school performance, sex, and so on). Alcohol abuse can cause further disinhibition and may lead the patient to manifest physical aggression, especially towards the family, with 'crimes of passion' being the most tragic result. The drug abuse pattern of BD patients tends toward the abuse of stimulant drugs. Familial diathesis for mania is significantly associated with the abuse of alcohol and drugs and it is possible that there is a common familial-genetic diathesis for a subtype of bipolar I, alcohol and stimulant abuse [114].

The cognitive deficits of BD patients have not been studied adequately. However in contrast to the early Kraepelinian concept for a favourable functioning outcome, recent studies suggest there is a significant degree of psychosocial impairment even when patients are euthymic and report that only a minority achieve complete functional recovery [115-121]. Cognitive impairment is reported to exist in both BD-I and BD-II patients, although more so in the BD-I group and this is true even during the euthymic period. The cognitive deficit could be worse during the manic phase, but it is present during all phases of the illness [122,123]. However, when compared to patients with schizophrenia, BD patients demonstrate a lesser degree of deficits, particularly concerning premorbidity and current intelligence quotient and perhaps attention, verbal memory, verbal fluency and executive functions [120,124]. The pattern of the neurocognitive deficit implicates the prefrontal cortex and temporolimbic structures, especially the ventromedial areas as well as the amygdala and the hippocampus.

Mood disorders are characterised by a constellation of symptoms and signs. The terms 'depressed mood', 'anhedonia' and 'elevated mood' are central to the definition and diagnosis of these disorders. The possible theoretical classification of symptoms and signs into four inter-related categories (mood, thought, behaviour and somatic) plus one accompanying regulating dimension (speed) is shown in Table S1 (see Additional file 1).

## Mood

**Euthymia **refers to the normal range of mood, and the absence of any disorder.

**Mourning **refers to the experience of sadness as a consequence of a loss of a loved one. It includes, crying, sadness, preoccupation with the lost person and related memories.

**Depressed mood **means that the patient experiences a 'negative' and unpleasant affect, and in English and other western cultures and languages the words (or their linguistic equivalents) 'depressed', 'anguished', 'mournful', 'sad', 'anxious', and 'blue' are used. The word 'depressed' is increasingly used because of the greater level of information (partially because of the internet) the public has today on depression. The way and the words the patient uses to describe this experience depend on their cultural and educational background, and can focus on bodily function or on existential and interpersonal dysphoria and difficulties. Somatic issues are more prominent in milder cases, usually seen in the primary care setting in patients with anxious depression. These cases were considered to have 'masked' depression.

**Anhedonia **refers to the inability to experience normal emotions. Frequently, patients with anhedonia are incapable of even feeling the depressed affect and they can't even cry. The patient abandons activities that in the past were a source of joy, and gives up interest in life. Patients with more severe depression are indifferent even concerning their children or spouse and isolate themselves. The difference from the flat (blunted) affect seen in schizophrenia is that anhedonia is itself painful. As depression starts remitting, anhedonia is one of the first symptoms to remit.

The term **elevated mood **refers to a state of elation, overconfidence, and enjoyment, with the person being cheerful, laughing and making happy and expressive gestures. It is not always pathological.

**Euphoria **refers to a pathologically overelevated mood that is inappropriate to real events. It is considered to constitute the opposite pole of 'depressed mood', with 'normality' in the middle. It is interesting and important to note that experiencing euphoria is pleasant, thus patients are reluctant to receive treatment.

**Expansive mood **is a condition with the patient expressing their feelings without restraint and control, and behaviour is usually coloured by grandiose thoughts.

**Emotional lability **refers to unstable and rapidly changing emotions because of hyper-reactivity to environmental stimuli. It is not always pathological

**Irritable mood **is a state in which the person is easily annoyed by external stimuli and expresses anger and hostility at a low threshold. The presence of an irritable mood is often cause for misdiagnosis of the patient, especially in combination with lability and mixed states.

## Psychomotor disorder

**Flight of ideas **refers to an acceleration of the thinking processes, and it manifests itself through rapid speaking. Speech could be coherent and thoughts unusually sharp, however when speed is excessively high they both become incoherent and fragmented, with content changing abruptly. Associations could be based on rhyme or chance perceptions.

**Psychomotor acceleration **is considered to be the hallmark of mania, characterised by excessive activity that is goal directed, high energy and endurance as well as rapid, pressured speech.

In comparison, **psychomotor agitation **also refers to a mental and physical overactivity (pressured speech, restlessness, motor behaviour) usually accompanied by a feeling of an inner turmoil or severe anxiety, with the intensity being so great that in spite of the fact that the patient has normal state of arousal, most if not all of this activity is purposeless.

**Psychomotor slowing **means that the patient is inert and slow, both physically and mentally, but this does not always have an effect on overall performance although everything is performed with much effort.

When psychomotor slowing is excessive, then **psychomotor retardation **appears, and it includes reduction or disappearance of spontaneous motor activity, slumped posture and gaze, reduced and slow speech and great fatigue.

**Stupor **appears in younger patients when the psychomotor retardation is so extreme that they are unable to function even concerning basic everyday needs. In more severe cases, motoric immobility appears.

**Catatonia **is defined as a complex condition that can include diverse symptoms and signs such as motoric immobility or, on the contrary, excessive purposeless motor activity not influenced by external stimuli, motiveless negativism, mutism, peculiar or stereotyped movements, mannerisms, grimacing and sometimes echolalia or echopraxia.

**Fatigue **is a common problem in all mental disorders but especially in mood disorders, and includes feeling tired or weak, sleepy, and sometimes irritable.

## Neurocognitive disorder

The term **neurocognitive **is often used with reference to higher cognitive function, such as attention, concentration, memory, praxis, and so on, and in psychiatry in contrast to the term **'**cognitive', which often is used with reference to thought content or style and relates to cognitive therapy. Bipolar patients constitute a clinically heterogeneous group, however they seem to perform poorly on most neuropsychological tests in comparison to healthy controls. They seem to have deficits especially related to attention, inhibitory control, spatial working memory, semantic verbal fluency, verbal learning and memory and maybe executive function, especially when considering the more severe and psychotic end of the bipolar spectrum. Verbal memory and likely executive function impairments may represent a trait rather than a state marker [119,125].

In extreme cases, neurocognitive disorder is so severe, especially in older patients, that the picture resembles that of a dementing disease; thus is called **pseudodementia**. However, it seems that at least half of these patients do in fact have a dementing process at its early stages and later they manifest a formal dementia syndrome [126-130]. If one looks at the problem from another point of view, depression with mild cognitive disorder may be either the first manifestation or a risk factor for the development of dementia, especially when combined with a family history of dementia [131-133].

## Thought disorder

**Depressive thought content **refers to depressed patients characterised by a negative evaluation of the self, the world, and the future (the negative cognitive triad). In this sense, the depressive thought content includes pessimism, low self-esteem and low self-confidence, ideas of loss, deprivation and guilt, helplessness and hopelessness, and ultimately thoughts of death and suicide. The extent to which this negative way of thinking is primary or secondary is a matter of debate.

**Clang association **refers to the condition when the patient's thoughts association and subsequently speech are directed by the sound of a word rather than by its meaning. Thus, words are not connected in a logical way and punning and rhyming serve as the drive.

**Thoughts of guilt **concern self-reproach, self-accusation and feeling the need for punishment. Thoughts and feelings of guilt are largely normal and they could appear during a mood disorder because of the disability the disorder causes and the inability of the patient to fulfil his/her obligations towards significant others. In this sense patients might also feel shame. However, when the intensity and the content is excessive or even inappropriate then thoughts of guilt should be considered to be part of the symptoms, and in more severe cases these thoughts could obtain a delusional character.

**Thoughts of death **are particularly important because they might eventually lead to suicidal behaviour. The common belief that inquiring about such thoughts provokes suicidal behaviour has no scientific basis. On the contrary, patients are often relieved. These thoughts include thoughts that the person will die and often they wish to die in some way so as to 'leave their suffering behind'; in this way, they lead to suicidal ideation.

**Suicidal ideation **refers specifically to thoughts of killing oneself. It has many different forms, ranging from indirect expression (for example, in a wish not to wake up or to die from a disease or an accident), to suicidal obsessions (urges or impulses to destroy oneself) and finally to elaborate planning of suicide. Some patients behave in a passive self-destructing way (for example, careless driving or walking), while others plan their death in detail leaving notes and making sure no help will come in time.

**Manic thinking **is excessively positive and optimistic. It is characterised by inflated self-esteem, a grandiose sense (concerning importance, power, knowledge, or identity), overconfidence and a sense of high achievement and abilities. Manic patients are refractory to explanations, confrontation and to a significant extent they lack self-examination and insight; because of this lack of insight, mania nearly always, sooner or later, acquires a delusional character.

## Psychotic symptoms

Psychotic features include delusions and hallucinations, and both can be mood congruent or non-congruent depending on their content. Mood-congruent psychotic features include those entirely consistent with thought content (either manic or depressive), while mood-incongruent features are largely unrelated to it. Psychotic features are not uncommon in mood disorders, especially in bipolar disorder, and delusions are relatively more common than hallucinations.

**Mood-congruent depressive delusions **are where depressed thoughts acquire a delusional severity and delusions congruent with depressive mood appear. Their content concerns inappropriate or overexaggerated thoughts of guilt, sin, worthlessness, poverty and somatic health. Nihilistic delusions constitute a special category under which the patient believes that parts of his/her body are missing. Delusions concerning persecution and jealousy, although seemingly non-congruent, could be mood congruent also, if they can be explained by or strongly related to thoughts of sin, guilt, jealousy or worthlessness. This kind of delusional thought makes a parent kill his/her family so as to save them from moral or physical corruption, and then he/she commits suicide.

**Nihilistic delusions **(Cotard delusion or Cotard's syndrome, negation delusion) are a special kind of delusion related to depressive mood and concern the delusional belief that all or parts of the patient's body are missing or rotten or decomposing, their internal organs are rotten or solidifying or even are actually dead; the world and everything related to it have ceased to exist.

**Mood-congruent manic delusions **are where, during manic episodes, the thought content usually becomes delusional, and include delusions of exceptional mental and physical fitness or special talents. It also may include delusions of wealth, and some kind of grandiose identity or importance. Sometimes the delusion can be so excessive that the identity itself changes (for example, the patient believes that he is a reincarnation of a messiah or a prophet, and so on). Delusions of reference and persecution are considered to be mood congruent on the basis of the belief that jealousy of the others at their special abilities is the cause of problems.

**Mood-incongruent delusions.** Various delusional ideas that are seemingly non-congruent (for example, ideas of persecution or reference) could eventually be understood as arising from a grandiose sense of self and the belief of the patient that this importance causes envy in others. However, sometimes there are delusions whose content has no association to current mood (for example, bizarre delusions without contextual relationship to mood). Sometimes a mixed mood episode can manifest itself with 'mood-incongruent' delusions (for example, grandiose delusions in the presence of depressed mood).

**Depressive mood-congruent hallucinations **are hallucinations whose content is consistent with either a depressed (for example, accusing or humiliating voices) or manic mood (for example, praising voices). Depressive mood-congruent hallucinations have an unpleasant content and they cause significant additional distress to the patient. Sometimes they command the patient to commit suicide and even dictate the method.

**Manic mood-congruent hallucinations. ** Sometimes it is considered that the intense experience of a mood episode, especially a manic one, causes such a vivid internal experience that the patients feel they can hear or see their thoughts (for example, hearing hymns or living in paradise).

**Mood-incongruent hallucinations.** These are hallucinations unrelated to the current mood state.

**Insight.** Classically, depressive episodes are characterised by a fair degree of insight with the exception of severe psychotic cases. In contrast, manic episodes are routinely characterised by a significant lack of insight and thus clinicians must routinely obtain basic information from others (relatives, friends, partners). This lack of insight might lead to refusal of any treatment and to the need for an involuntary admission to a hospital.

## Somatic and neurovegetative symptoms

Depressed patients often manifest changes in appetite, sleep and sexual functioning. Circadian rhythms are also disrupted. The classical notion of depression, which is closer to melancholia, includes reduction in all these functions; however, recently the 'atypical' form of depression was described and this form includes an increase in these neurovegetative functions; that is, overeating and oversleeping, along with interpersonal rejection sensitivity, which is a 'personality-like' feature.

**Anorexia and weight loss **are considered to be reliable signs of depression. They can both be considered in the sense of a generalised inability to enjoy things (anhedonia). Weight loss is sometimes seen in paranoid patients who are afraid that food is poisoned, and this should not be confused with anorexia and weight loss in the frame of depression. Weight loss is also frequent in cases of malignant disease, so a full medical investigation should be given to any patient with changes in appetite or weight.

**Weight gain **has been relatively recently recognised as a depressive feature, and could be the result of overeating, decreased activity, or both. Apart from its devastating effect on self-confidence and self-image, it can worsen general somatic health, especially in patients that become obese and have metabolic syndrome.

**Insomnia **is one of the hallmarks of depression and one of its most disturbing features. There are many types of insomnia, such as difficulty falling asleep (initial insomnia), multiple awakenings during the night (middle insomnia) or early morning awakening (terminal insomnia). Insomnia prolongs the depressive agony round the clock. Some patients try to self-medicate and solve the problem by alcohol or drug abuse (sedatives or hypnotics), but both eventually worsen the problem partially because of tolerance and dependence problems and partially because they both further destroy the architecture of sleep. Unipolar depressed patients stereotypically tend to exhibit insomnia episode after episode and characteristically, in spite of extreme fatigue, they rarely oversleep.

**Hyposomnia **is a decreased need for sleep. That is, the patient feels energetic on awakening even though he or she only slept for a short period. Some patients feel fresh and energetic even though they haven't slept for days. This condition is usually seen during manic episodes, and sometimes it heralds the beginning of such an episode.

**Hypersomnia.** Some patients especially younger ones and females often sleep too much and find it difficult to get up in the morning. Along with the other atypical features it is considered to be a marker for an underlying bipolar illness even in cases no other bipolar feature is present. This condition should be differentially diagnosed from a number of medical conditions including narcolepsy and the Klein-Levin syndrome. In spite of prolonged sleep, depressed patients are characteristically tired in the morning, meaning that even prolonged sleep is not refreshing for them. The change in the pattern of sleep disruption with insomnia alternating with hypersomnia or hyposomnia suggests the presence of a bipolar illness rather than a unipolar depression.

**Circadian dysregulation. **Although many circadian functions could be disrupted in depressed patients, mainly the disturbance of sleep rhythms has been adequately studied. This disturbance includes deficits in delta sleep and more intense rapid eye movement (REM) activity during the first third of the night. A marked shortening of REM latency (that is the time from the onset of sleep to the first REM period) is considered to be characteristic for depression of any type, and seen even in remitted depressive patients and their healthy relatives.

**Seasonality,** Seasonal (especially autumn and winter) emergence or worsening of depression has been recognised since antiquity, and mood has been related to the period of the year. Most patients seem to experience increased energy and activation during spring and the opposite during the autumn and winter. Usually, patients with strong seasonality also have reverse neurovegetative symptoms (fatigue, craving for sugars, overeating and oversleeping). In some patients seasonality is so concrete and important that modern classification includes a seasonal pattern for mood disorders.

**Sexual dysfunction.** Depressed patients classically report a decreased sexual desire and activity, while additionally some women manifest a temporary interruption of their menses. Sexual dysfunction especially in females could lead to marital conflict, and a psychodynamic/psychotherapeutically-oriented therapist could mistakenly ascribe depression to the marital conflict with profound negative effects on the therapeutic outcome. Treating the sexual dysfunction or its consequences and leaving depression untreated is not uncommon and includes even surgical or unusual therapeutic interventions. An additional problem is that treatment with antidepressants often has sexual dysfunction as an adverse effect. The recent emergence of agents that treat impotence (for example, sildenafil, tadalafil) could add a new method to treat this problematic symptom, but this should never move the focus of treatment away from depression.

**Increased sexual desire and activity **is typical for manic episodes, but also a subgroup of depressed patients may manifest increased sexual drive or activity and usually they also manifest other atypical or 'reversed' features. Thus, if seen in the frame of depression it heralds the presence of a depressive mixed episode. The increased sexual appetite usually leads to sexual indiscretion accompanied by a risky sexual life, often leading to marital problems, multiple separations or divorce, alcohol and drug abuse, gambling and sexually transmitted diseases such as AIDS.

## Behavioural disorder

**Logorrhoea **refers to pressured, excessive and not always coherent speech, which is often uncontrollable. It is observed during manic episodes. Speech could be completely uncomprehending, with destroyed syntax and loose associations, often posing diagnostic dilemmas (for example, from stroke). Other similar terms used are tachylogia, verbomania and volubility.

**Impulsive behaviour.** During mood episodes (manic, depressive or mixed), patients tend to exhibit impulsive behaviour. In particular during manic episodes, they tend to be impulsive, disinhibited and meddlesome. They are intrusive, with increased involvement with others, have poor social judgment and engage in a variety of activities without control or restraint (including aggression, sex, gambling, drug and alcohol abuse, overspending, making gifts, risk taking, travelling, and so on). Impulsive behaviour is the part of symptomatology that causes the most problems, in particular financial and interpersonal ones. In some cases even suicide could be attempted on an impulsive basis.

The terms 'endogenous depression', 'neurotic depression', 'anxious depression', 'involutional melancholia' and 'psychotic depressive reaction' are not included in modern classification systems for a variety of reasons. The term 'neurasthenia' is maintained in ICD-10, but its meaning is vague.

It seems that the psychotic melancholic subtype is the most stable type of depression, repeating itself across episodes [134]. Almost a third of all major depressive episodes do not recur and it seems that recurrent depression is more familial, with an average episode duration of 6 months and a varying interepisode interval length. A significant proportion of patients remain symptomatic and disabled, many of them with subsyndromal depression [135]. Around 15% develop psychotic features.

## Comorbidity

Large epidemiological studies and clinical experience suggest that mood disorders either coexist or overlap considerably with anxiety disorders. Anxiety disorders can occur during a depressive episode, may be a precursor to it or may appear during the future course of a mood disorder. Several authors suggest there is a common diathesis connecting mood and anxiety disorders, with more recent data suggesting a strong link between BD-II and panic, obsessive-compulsive behaviour, and social phobia.

Somatic illness frequently coexists with depression and anxiety, and the mood disorder has a profound negative impact on the outcome of the somatic illness. The therapist should also suspect clinical depression in all patients who refuse to participate in medical care.

All mood disorders, but especially bipolar disorder, are highly likely to be comorbid with alcohol and drug (mainly stimulant) abuse, usually in the sense of a self-treatment effort from the side of the patient [114]. A large variety of different substances can be related with use or abuse, and consequently with substance-induced mood disorders [114,136]. They include various medications (for example, anaesthetics, anticholinergics, antidepressants, anticonvulsants, antibiotics, antihypertensives, corticosteroids, antiparkinsonism agents, chemotherapeutic agents, non-steroidal anti-inflammatory drugs, and disulfiram), toxic agents (heavy metals, industrial solvents, household cleaning agents), or substances used routinely for recreational purposes (for example, caffeine, nicotine). Almost all the substances are preferred because of their subjective effects, which concern mainly mood. Others are used for their calming or 'therapeutic-like' effects (as self-treatment; for example, alcohol and sedatives) while others for their stimulating, euphoric and augmenting effects (for example, stimulants).

Substance use and abuse could happen in the frame of a pre-existing mood disorder or the use itself can be the cause of the disorder because of the direct physiological effects (toxicosis, withdrawal or dependence). When the mood disorder is primary and pre-exists, substance use complicates both the clinical manifestations and the treatment, and might lead to poor prognosis. This is especially true during the teenage and early adult years, relates mainly to cyclothymia and probably represents attempts at self-medication for the mood liability. During the withdrawal period many substances including alcohol, opioids, and sedatives might induce persistent mood disturbance, insomnia and cognitive disorder, leading to relapse of the abuse. These symptoms need to be distinguished from those of primary mental disorders, and this is often very difficult. The critical factor is the clinician's judgment of whether the mood disorder is caused by the substance or not. A double diagnosis is usually the only reasonable solution. However, the 'self-medication' scenario, with mood disorder being the primary diagnosis, or even part of a double diagnosis, is unfortunately not the diagnostic priority of many therapists (especially in therapeutic communities), and consequently the diagnosis of mood disorder is missed, depriving the patient of proper and effective treatment.

Alcohol use and abuse is very frequent, especially for mood and anxiety disorder patients. However, heavy alcohol consumption over a period of days results in a depressive state, which, even when it is severe, largely improves within days to weeks of abstinence. After several weeks, most alcoholic patients manifest residual low mood or mood swings resembling a cyclothymic or dysthymic disorder, but they also tend to diminish and disappear with time. The presence of the dysthymic symptoms usually indicates the normal course of a withdrawal syndrome and not an independent mood disorder. Nicotine use and abuse is also very frequent, usually in the form of cigarette smoking, and withdrawal is manifested by changes in mood, anxiety and weight gain (average is 2 to 3 kg), which can persist for months.

Amphetamine, cocaine, opioid, hallucinogen or inhalant-induced mood disorder can occur during intoxication or withdrawal. In general, for all these substances, intoxication is associated with manic or mixed mood features, whereas withdrawal is associated with depressive mood features. An induced mood disorder by any of them usually remits within 1 to 2 weeks (several weeks for opioids), except for panic episodes that develop during cocaine use that can persist for many months following cessation [137,138]. An important outcome is suicide, which is not an uncommon event.

## Classification

The ICD-10 includes sets of criteria for mood disorders that are used throughout the world and constitute the official method of reporting health statistics. They overlap with the DSM-IV-TR criteria; however, important differences do exist.

The basis of the classification in both systems is the definition of the depressive and manic/hypomanic episodes. A novel classification for bipolar disorder, by utilising the concept of the bipolar spectrum ranging from bipolar 0 (schizophrenia) or bipolar 1/2 (schizoaffective disorder) to bipolar VI (bipolarity in the frame of dementia) is shown in Table 1[107,139,140].

**Table 1:** Bipolar disorder (BD) types, from BD-0 to BD-6

BD type	Description
BD-0	Schizophrenia
BD-1/2	Schizobipolar disorder
BD-I	'Classic' bipolar disorder
BD-I1/2	Depression with protracted hypomania
BD-II	Hypomania plus major depression
BD-II1/2	Depression superimposed on cyclothymic temperament
BD-III	Recurrent depression, plus hypomania occurring solely in association with antidepressant or other somatotherapy
BD-III1/2	Mood swings that persist beyond stimulant and/or alcohol abuse
BD-IV	Depression superimposed on a hyperthymic temperament
BD-V	Recurrent depressions without discrete hypomania, but mixed hypomanic episodes (irritability/agitation/racing thoughts) during depression
BD-VI	Bipolarity in the frame of dementia

## Differential diagnosis

Mood disorders should be differentially diagnosed from a number of other morbid conditions, both psychiatric and non-psychiatric.

Several mental disorders, including alcohol and substance use disorders, normal bereavement, depression in the frame of schizophrenia, anxiety disorders, personality disorders, dementia and a variety of general medical conditions that cause syndromes similar to depression, should be differentiated from mood disorders. Also, several drugs used for the treatment for a number of diseases might also cause depression. In general, the prevailing opinion is that a missed diagnosis of mood disorder in favour of another mental diagnosis might mean that the patient does not receive proper treatment, which can have serious consequences.

Perhaps the most important differential diagnosis should be made between mood and personality disorders. Since the state dependency of most personality features is well documented [141-149], clinicians should avoid putting this diagnosis in patients with an active mood disorder, even in cases where this mood disorder is subthreshold. A dangerous stereotypical thinking leads clinicians to suggest that, because a patient does not respond adequately to usual treatment, the disorder is personality based. This is especially problematic concerning subthreshold or non-classic mixed clinical pictures, which are relatively refractory to treatment and cause issues for the therapist.

Normal bereavement appears normally in persons experiencing the loss of a significant other, and consists of several depressive symptoms during the first 1 to 2 years after the loss. But only around 5% will eventually progress to a depressive disorder. Normal bereavement is generally contrasted with depression because reactivity to environmental stimuli is preserved, the disability if any is mild, and no severe psychopathology (delusions or hallucination or true suicidal ideation) is present.

Anxiety symptoms commonly occur in mood patients, including panic attacks, fears, and obsessions. Longitudinal data suggest that although the depressive symptoms tend to remit in time, the anxiety symptoms persist. Because anxiety disorders rarely appear for the first time after the age of 40, a late appearance of significant anxiety should be considered to be a sign of depression. Transient and periodic monosymptomatic phobic and obsessional states that do not fulfil criteria for a formal disorder as conceptualised in either classification system should also be considered as reflecting an underlying mood disorder, and should be treated accordingly.

Somatic issues especially in depression might also reflect an underlying physical illness rather than a somatisation mechanism. The somatic disorders most commonly related to depression are multiple sclerosis, Parkinson's disease, head trauma, epilepsy, sleep apnoea, cerebral tumours, vascular encephalopathy, chronic fatigue syndrome, some collagen disorders such as rheumatoid arthritis and lupus erythematosus, and various neoplastic conditions such as abdominal malignancies (especially in the pancreas) and disseminated carcinomatosis. Also, there are a number of abnormal endocrine conditions including hypothyroidism and hyperthyroidism, hyperparathyroidism, hypopituitarism, Addison's disease, Cushing's disease and diabetes mellitus, several infections such as general paresis (tertiary syphilis), toxoplasmosis, influenza, viral pneumonia, viral hepatitis, infectious mononucleosis and AIDS, and nutritional conditions such as pellagra and pernicious anaemia.

A number of pharmacological agents used for the treatment of various diseases can cause depression or a depressive-like condition. These include α-methyldopa, anticholinesterase insecticides, cimetidine, cycloserine, indomethacin, mercury, phenothiazine antipsychotic drugs, reserpine, steroidal contraceptives, thallium, vinblastine and vincristine.

Withdrawal from agents such as amphetamine, alcohol or sedative hypnotics can also be the cause of depression.

In geriatric patients, the differentiation between depressive pseudodementia and degenerative dementia is vital and is made via a neuropsychological profile of the patient as well as from the clinical course, which in pseudodementia cases includes an acute onset without prior cognitive disorder, a personal or family history of affective illness, circumscribed memory deficits and an unstable cognitive dysfunction that can be reversed with proper coaching.

The need for the differential diagnosis of mood disorders from the above-mentioned conditions makes it important for the clinician to obtain a variety of laboratory examination data, including standard blood and biochemical tests, EEGs, electrocardiograms (ECGs), thyroid function tests and (depending on availability and cost) even brain magnetic resonance imaging (MRI) scans, and in late onset cases indices assessing malignancy.

## Suicide

Today we know that suicide is a complex and multicausal behaviour and demands a complex and sophisticated approach. Statistics point to a substantial decline of suicide rates throughout Europe, the US and Canada over the past two decades, and the major reason for that seems to be the better recognition of major depression as well as availability of treatment [150-154]. The understanding and prevention of suicide is one of the most challenging tasks for psychiatry today. It has been confirmed by several psychological autopsy studies that the majority of suicidal victims had a mood disorder, usually untreated major depression, with frequent comorbidity of anxiety and substance use disorders [154-159]. Around 60 to 80% of all suicide victims have depression, while in contrast an estimated 15% of patients with severe major depression eventually die from suicide. The ratio of attempted to completed suicide, is about 5:1 in patients with any mood disorder [160].

Although many risk factors have been identified, most of them are not clinically useful. An important and useful risk factor is the presence of a depressive mixed state (three or more simultaneously co-occurring hypomanic symptoms in patients with 'unipolar depression'). This clinical picture overlaps to a great extent with agitated depression. Depressive mixed state as well as agitation substantially increase the risk of both attempted and committed suicide [150-152,156,161]. Other risk factors include family history of suicide, higher number of prior depressive episodes, comorbid anxiety, personality disorders and alcohol dependence, as well as sociodemographic and psychosocial factors such as younger age, being divorced or widowed, and experiencing adverse life situations that are associated with increased suicidal ideation and higher prevalence of attempts [151,154-156,161,162]. Although biological research has so far identified several biological correlates of suicide, to date there is no biological marker to distinguish explicitly between suicidal and non-suicidal depressives [163,164].

An important fact is that in spite of frequent medical contact before committing suicide, only a small minority of victims had received appropriate treatment. This is particularly a problem in primary care, where most patients seek help [154,155,165-167]. Thus, not only is early identification of suicidal behaviour possible, but also early intervention is possible and could make a difference. The patient should be put on a plan of regular psychiatric visits on an interval ranging from once to twice weekly. Latter visits could be planned on a monthly interval or even less frequently. The main factors determining frequency include the clinical picture, social and family support, history of adherence, insight into the illness and the risk and adverse effects of medication. The therapist should have in mind that antidepressive agents are the only formally approved treatment for major depression [151,152,168] and there are no data supporting the effectiveness of any other approach [169]. Also, a marked antisuicidal effect has been also reported with long-term lithium therapy in bipolar (manic depressive) patients [151,153,170].

Recently, the US Food and Drug Administration issued a warning concerning the use of antidepressants in children and adolescents and possibly in all age groups because of possible induction of suicidality (thinking and behaviour but not completed suicide) by antidepressants in juvenile depressives [171]. A similar warning is now in place concerning anticonvulsants. However, the impact of this warning might be robustly negative.

The warnings are based on data from randomised controlled trials (RCTs), but there is doubt whether the design of these studies permits these conclusions. A recent study reports that after the warning, (between 2003 and 2005) the selective serotonin reuptake inhibitor (SSRI) prescriptions for children and adolescents in the US and The Netherlands decreased by about 22% but simultaneously there was a 49% youth suicide rate increase in The Netherlands (between 2003 and 2005) and a 14% increase in the US (between 2003 and 2004) [172]. It is highly possible the 'natural' population of mood disorder patients does not respond to treatment in this way. However, it seems that proper and 'aggressive' treatment of mental disorders and especially of major depression aiming at achieving full remission should always be the target and determines to a large extent whether suicidal behaviour is expressed or not [170,173-175]. A caveat is that the most dangerous period for suicide in a patient is immediately after treatment has commenced, as antidepressants may reduce the symptoms of depression such as psychomotor retardation or lack of motivation before mood starts to improve. Although this appears to be a paradox, studies indicate the suicidal ideation is a relatively common component of the initial phases of improvement even with psychotherapy [176].

## The age effect

Although the core features of mood disorders are essentially the same across a lifetime, traditionally children and older patients are considered somewhat separately because of the special features their phases of life include, and the way these features might influence the overall manifestation of mental disorders and their treatment. Additionally, an early age of onset of any disorder puts forward the question whether this determines a more severe and chronic disease and also poor response to treatment. The ratio of males to females with MDD remains stable across the age spectrum [177].

It seems that the developmental phase might influence the expression of certain mood symptoms and this is why, for example, pervasive anhedonia or significant psychomotor retardation are rare among depressive children and auditory hallucinations and somatic issues are seen more often in prepubertal children.

The incidence of mood disorders among children and adolescents is reported to have increased during the last few decades. These reports are consistent and they also suggest there is a decrease of the age of onset of mood disorders. The general picture suggests that the prevalence of depression is around 0.3% for preschool children, 0.4% to 3% for school age children and 0.4% to 6.4% for adolescents; the prevalence of bipolar disorder is 0.2% to 0.4% in children and 1% in adolescents. Research suggests that 40% to 70% of children and adolescents with a mood disorder also have at least one comorbid psychiatric disorder. It also seems that childhood depression is prebipolar in the majority of cases [178]. The risk factors as well as the etiopathogenesis for this age group are uncertain.

A worldwide trend is the increase in both the absolute numbers and percentages in the total population of older people. This of course leads to an increase in the number of geriatric psychiatric patients and a shift of the focus of healthcare services. At the same time, geriatric mental patients present with multiple challenges both at the diagnostic as well as the therapeutic level. The prevalence of major depression is estimated to be 2% in the general population over 65 years of age [179-181], with up to 15% having some kind of other mood disorder [182] and 25% to 40% of patients in the general hospital setting having a subthreshold depression [183]. In residential homes, the accepted value for patients with MDD is approximately 12%, with an additional 30% manifesting a milder form of depressive-like symptomatology [184-189]. The recognition of geriatric mood patients (with a late onset mood disorder) is poor, and less than 50% of hospitalised patients with depression in general medical practice are referred to a psychiatrist, and less than 20% receive adequate treatment [190].

With regard to suicide and related behaviours, the attempted suicide rate is 1% in children and 1.7% to 5.9% in adolescents, while the completed suicide rate ranges from nearly 0 in children below the age of 10, to a peak of above 18 in 100,000 in boys 15 to 19 years old. The data suggest that among 15 to 19 year olds, the suicide rates have quadrupled over the last four decades, and the reason for this is not known. Unfortunately, suicide is currently the fourth leading cause of death in children aged 10 to 15 years and the third leading cause of death among adolescents and young adults aged 15 to 25 years. The suicide method is the most significant factor in determining whether the attempt will result in death. The great majority of attempts among children and adolescents have little lethal potential, partially because of restricted access to lethal material and inadequate cognitive potential to plan a successful attempt. What is unique in this age group is suicide imitation and contagion. This means that the suicidal behaviour increases in adolescents following exposure to well publicised news stories of suicide or a film involving a teen suicide, but this seems to concern vulnerable individuals and not the age group as a whole [191-193]. At the same time, geriatric patients with depression have up to 1.5 to 3 times higher morbidity [194], with the lifetime risk of suicide being as high as 15%; almost 10% of them die annually [195].

The etiopathogenesis of mood disorders in children and adolescents is not well understood. It is an age group that combines developmental vulnerability and high potency for neuroplasticity and compensation for any insults. It is generally believed that genetic factors play a significant role, however there are vague data in support of this and no clear conclusions can be made. Non-shared environmental factors might also play an important role [196]. At the cognitive level, the theoretical approach suggests the presence of cognitive distortions similar to those seen in adults, but again data are inconsistent and scarce.

Traditionally there has been significant interest on the family interactions and their relationship to the development of depression, but the conditions are usually complicated and difficult to interpret. The most difficult problem is that when the family environment is problematic, there is a high probability of a genetic vulnerability in the family and sometimes in both parents. However this does not exclude the possibility that the environment can induce a kind of emotional vulnerability in the child by shaping the early experiences. Depressed parents may model negative cognitive styles and poor self-esteem, leading to a deficit of social problem-solving skills and ability in coping with stressful life events; marital conflict and lack of an adequate family support system, especially when a mental illness of the parent(s) of an early onset is recurrent, and disruption of parental functioning, put the child at a high risk for any mental disorder but especially for a mood disorder. In this sense, it is understandable why family conflict is the most frequent event adolescents report they have experienced before they manifest suicidal behaviour. There are several studies suggesting that depressed children and adolescents might experience more stressful life events such as interpersonal losses, problems in relationships, parental divorce, bereavement, physical abuse and suicide in the environment [197-200].

The conclusion concerning the etiopathogenesis of mood disorders in children and adolescents is that genetics clearly plays at least a moderate role, while both shared and non-shared environmental influences appear to also be important.

However, late onset mood patients are less likely to have a positive family history for mood disorders compared to younger patients [201,202] and are more likely to manifest structural changes of the CNS [177,203,204]. Neuroimaging studies have reported a variety of morphological disturbances, which clearly differentiate late-life depression from depression of younger ages [204-210], suggesting an association to an increased severity of subcortical vascular disease and greater impairment of cognitive performance [211]. Moreover, major depression is more common and more severe in patients with vascular dementia [212].

Clinically, depression in children and adolescents presents with the same core features manifested in adults. Some minor differences suggest the presence of irritable rather than depressed mood, and failure to attain expected weight gain instead of weight loss. Among preschool children, lack of smiling, apathy towards play, lack of involvement in all activities, physical issues, and physical aggression are common signs, while among school-aged children, deteriorating school performance, increased irritability, fighting, or argumentativeness and avoidance of peers may signal depression. Exacerbation of anxiety symptoms and refusal to attend school are not uncommon among children who are depressed. Switching from unipolar depression to bipolar disorder is significantly higher in children than it is in adults, and it reaches 32% within a 5-year period. Also, it is reported that in children, mania might present with a chronic instead of an episodic pattern, with mixed and rapid cycling features instead of classic manifestations and highly comorbid mental disorders. These suggest that childhood-onset bipolar disorder is a more severe form of the illness, and relatively treatment resistant. The main disorders that should be differentially diagnosed are attention deficit hyperactivity disorder (ADHD) and disruptive behaviour disorders [213].

Various studies of depression in older people have reported that mood is more often irritable than depressive [214], and also several symptoms such as loss of weight, feelings of guilt, suicidal ideation, melancholic features, hypochondriasis as well as associated symptoms of psychosis can be more frequent [215-219]. However, these findings vary across studies. Many of these patients manifest a type of behaviour that can be characterised as 'passive-aggressive' or 'self-aggressive'. They refuse to get up from bed, eat, wash themselves, or talk. Also, they often hide important information concerning severe somatic disease and in this way they let it go untreated.

Somatic symptoms are difficult to assess and, as a general rule, doctors should avoid assigning this symptomatology to an underlying mental disorder. It is highly likely the patient indeed has a true 'somatic' disorder even in cases where the doctor is unable to diagnose it [220]. However, it is clear that older depressives manifest more somatoform symptomatology, in comparison to younger depressives. In this sense, the concept of Masked Depression [221] used to be popular in the past, but today it is not accepted by either classification system although it is accepted that the onset of health concerns in old age is more likely to be either realistic or to reflect a mood disorder [220]. Percentages of comorbidity between depression and physical illness vary from 6% to 45% [222,223]. The large discrepancy reflects the difficulty in the application of operationalised criteria for the diagnosis of depression in patients with general health problems. Greater overall severity of medical illness, cognitive impairment, physical disability and symptoms of pain or other somatic issues seem to be a more important predictors of depression than specific medical diagnoses [224].

About 38% to 58% [225] of older people with major depression also fulfil criteria for an anxiety disorder, while many authors have suggested that the presence of anxiety in older people should be considered as a sign of depression, even in cases, which lack true depressive symptomatology [226].

In older individuals there is an increased possibility of the coexistence of depression and dementia, or some other type of 'organic' decline of cognitive disorder. The syndrome of 'pseudodementia' has also been described [227]. This term refers to the manifestation of dementia symptomatology, which in fact is due to depression and disappears after antidepressant therapy. The emergence of late onset bipolarity in the sense of an ongoing dementing pathology has also been described [107,139,140].

Suicide constitutes an important health problem for older people. Older men are at a higher risk for completing suicide than older women. The coexistence of a serious somatic disease, such as renal failure or cancer, represents a major risk factor for a well planned suicide attempt [228]. Other risk factors include loneliness and social isolation, usually as a consequence of bereavement. Failure to follow medical advice in serious general medical conditions could be considered to be a form of 'passive suicide'. However, 'rational' suicide plans are not common even in severely ill patients. There is a possibility of acute-onset suicidal plans (after an acute incidence concerning general health, for example, stroke or heart attack) [229].

The psychological treatment of children and adolescents with mood disorders are similar to those for adults. However, there is a significant controversy concerning pharmacotherapy. Double-blind studies are missing and it seems that these age groups are particularly vulnerable for the induction of suicidality by antidepressants. Flouxetine, quetiapine and lithium are the better-studied agents in terms of efficacy in these age groups [230-240]. Electroconvulsive therapy (ECT) and transcranial magnetic stimulation (TMS) might be reasonable alternatives if initial therapeutic attempts fail [241].

The pharmacotherapy of late-onset mood disorder includes the cautious use of antidepressants including amitriptyline, imipramine, nortriptyline and the SSRIs, which are the most widely prescribed antidepressants among the geriatric population because of their favourable side-effect profile, relative safety in overdose, ease of use and smaller dosage adjustment, making them first-line choices. Also, venlafaxine, mirtazapine, and bupropion can be useful.

For bipolar cases, lithium and anticonvulsants are useful, although they are not well studied in older patients [242]. They are mostly used in cases of refractory depression for the augmentation of antidepressant therapy. Antipsychotics, especially second generation ones, could be used although there is a warning for a higher mortality because of their use in older patients. ECT is another option with many studies reporting better outcomes in older than in younger patients. However by far the most troubling side effect of ECT, especially in older people, is cognitive impairment.

Psychotherapy is also an option [243,244]. The presence and severity of medical illnesses, physical disability, cognitive impairment and psychomotor retardation make psychotherapeutic intervention difficult and affect its efficacy and success. The form of psychotherapy should be adjusted to the patient's personality, behaviour patterns as well as his/her cultural and educational level. Behavioural therapy, cognitive behavioural therapy and problem-solving therapy have been extensively studied for their effectiveness in the treatment of depression in older people. Fewer studies have been carried out for the efficacy of interpersonal psychotherapy. Non-standardised psychotherapies, such as psychodynamic psychotherapy and reminiscence therapy, are also proposed as appropriate treatments for geriatric depression.

Combination of pharmacological and psychological treatment is associated with higher improvement rates than pharmacotherapy alone, and considered more effective than either treatment alone in preventing recurrence of depression [245]. In long-term therapies, the addition of psychotherapy promotes adherence to treatment [246].

Eventually, however, most studies support the opinion that geriatric depression carries a poorer prognosis than depression in younger patients. However, many authors attribute this to factors such as failure to make an early diagnosis and improper or insufficient treatment. For patients with geriatric depression, the prognosis is more dependent on physical handicap or illness and lack of social support, however further research on this issue is needed. Thus, the effective prevention of late-life depression requires attention to maintaining community infrastructure and support.

## Competing interests

KNF is member of the International Consultation Board of Wyeth for desvenlafaxine and Solvey for agomelatine, and the Greek board of BMS for bipolar disorder and has received honoraria for lectures from AstraZeneca, Janssen-Cilag, Eli-Lilly and research grants from AstraZeneca, Wyeth and the Pfizer Foundation

## Appendix

### Appendix 1

Prognostic elements for a future manic or hypomanic episode in (pseudounipolar) depressed patients.

• Very early onset (childhood or early adolescence).

• Cyclothymic temperament.

• Any cycling at all (for example, between 'somewhat depressed' and 'severely depressed'): emotional liability.

• Type A behaviour (especially the S-factor; that is hurried, quick tempered, irritable).

• Mood-incongruent behaviour.

• The presence of even a single symptom of the opposite pole.

• Induction of the opposite pole by classical antipsychotics or antidepressants.

• Psychotic disorder family history.

## Supplementary Material

Additional file 1 **Supplementary table 1**. List of mood symptoms and signs as they might intercorrelate or correspond to each other, classified in four domains (mood, thought, behaviour and somatic) plus one regulatory dimension (speed). Click here for file
