# Supplementary material for: The emerging modern face of mood disorders: a didactic editorial with a detailed presentation of data and definitions
Source: Ann Gen Psychiatry. 2010 Apr 12;9:14. doi: 10.1186/1744-859X-9-14 (PMC2865463; doi:10.1186/1744-859X-9-14)
Supplement: Additional file 1 — Supplementary table 1. List of mood symptoms and signs as they might intercorrelate or correspond to each other, classified in four domains (mood, thought, behaviour and somatic) plus one regulatory dimension (speed). [file 1744-859X-9-14-S1.DOC]

**Table S1.** List of mood symptoms and signs as they might intercorrelate or correspond to each other, classified in four domains (mood, thought, behaviour and somatic) plus one regulatory dimension (speed)

| **Depressive episode** | | | | | **Manic episode** | | | | |
| --- | --- | --- | --- | --- | --- | --- | --- | --- | --- |
| **Mood** | **Thought** | **Behaviour** | **Somatic** | **Speed** | **Mood** | **Thought** | **Behaviour** | **Somatic** | **Speed** |
| Depressed mood | Low self-esteem |  |  | Variable | Mood elevation | Inflated self-esteem or grandiosity |  |  | Variable |
| Reduced reactivity of mood |  | Passive behaviour |  | Reduced | Irritability, anger |  | Verbal/physical aggressiveness |  | Increased |
| Anhedonia |  | Facial and body activity goal-directed activity |  | Reduced | Excessive involvement in pleasurable activities |  | Facial and body activity goal-directed activity |  | Increased |
| Mood liability | Variable | Variable |  | Variable | Mood liability | Variable | Variable |  | Variable |
|  | Ideas of guilt, sin | Introversion |  | Variable |  | Expansive attitude | Extraversion |  | Variable |
|  | Pessimism | Passive behaviour |  | Variable |  | Overoptimistic | Impulsivity |  | Variable |
|  | Concentration difficulties | Decreased speech |  | Reduced |  | Hyperconcentration, sharpened thoughts | More talkative than usual |  | Increased |
|  | Dull thinking | Decreased speech |  | Reduced |  | Unusually creative thinking | More talkative than usual |  | Increased |
|  | Inhibition of thoughts | Diminished speech |  | Reduced |  | Flight of ideas/racing thoughts | Pressure to keep talking, articulate and jocular |  | Increased |
|  | Indecisiveness |  |  | Variable |  | Distractibility |  |  | Variable |
|  | Concern | Reservation |  | Reduced |  | Disregard | Impulsivity |  | Increased |
|  |  |  | Insomnia/hypersomnia | Variable |  |  |  | Decreased need for sleep | Increased |
| Loss of libido | Lack of sexual thoughts | Reduced sexual activity | Impotence | Variable | Increased libido | Sexual fantasies | Increased sexual activity | Arousal | Increased |
|  |  |  | Tiredness/fatigue/leaden paralysis | Reduced |  |  |  | High energy levels, excessive endurance | Increased |
|  |  |  | Weight/appetite changes | Variable |  |  |  | Weight/appetite changes | Variable |
|  |  | Disorganised |  | Psychomotor retardation/agitation |  |  | Disorganised |  | Psychomotor agitation |
| Social anxiety | Low self-esteem | Psychosocial withdrawal, behavioural inhibition |  | Variable | Loss of social inhibitions, impulsive behaviour/disinhibition | Inflated self-esteem or grandiosity | Increased sociability |  | Increased |
|  | Hopelessness/helplessness, suicidal thoughts | Suicidal acts |  | Variable |  | Omnipotent thoughts | Reckless behaviour |  | Increased |
| Anxiety | Worries | Restlessness | Autonomic activation, gastrointestinal, various pain, other somatic symptoms | Variable | Anxiety | Worries | Restlessness | Autonomic activation | Variable |
| Anxiety | Concern | Reservation |  | Variable | Overconfidence | Disregard | Impulsivity |  | Increased |

Almost any combination is possible in real life clinical practice and especially in the frame of mixed not otherwise specified (NOS) mood episodes.
